# Incidence of cardiovascular events and associated risk factors in kidney transplant patients: a competing risks survival analysis

**DOI:** 10.1186/s12872-017-0505-6

**Published:** 2017-03-07

**Authors:** María Teresa Seoane-Pillado, Salvador Pita-Fernández, Francisco Valdés-Cañedo, Rocio Seijo-Bestilleiro, Sonia Pértega-Díaz, Constantino Fernández-Rivera, Ángel Alonso-Hernández, Cristina González-Martín, Vanesa Balboa-Barreiro

**Affiliations:** 1 Clinical Epidemiology and Biostatistics Research Group, Instituto de Investigación Biomédica de A Coruña (INIBIC), Complexo Hospitalario Universitario de A Coruña (CHUAC), SERGAS, Universidade da Coruña, Hotel de Pacientes 7ª Planta, C/As Xubias de Arriba, 84, 15006 A Coruña, Spain; 2 Department of Nephrology, A Coruña Hospital, As Xubias 84, A Coruña, 15006 Spain

**Keywords:** Kidney transplantation, Cardiovascular diseases, Risk factors, Survival analysis

## Abstract

**Background:**

The high prevalence of cardiovascular risk factors among the renal transplant population accounts for increased mortality. The aim of this study is to determine the incidence of cardiovascular events and factors associated with cardiovascular events in these patients.

**Methods:**

An observational ambispective follow-up study of renal transplant recipients (*n* = 2029) in the health district of A Coruña (Spain) during the period 1981–2011 was completed. Competing risk survival analysis methods were applied to estimate the cumulative incidence of developing cardiovascular events over time and to identify which characteristics were associated with the risk of these events.

Post-transplant cardiovascular events are defined as the presence of myocardial infarction, invasive coronary artery therapy, cerebral vascular events, new-onset angina, congestive heart failure, rhythm disturbances, peripheral vascular disease and cardiovascular disease and death. The cause of death was identified through the medical history and death certificate using ICD9 (390–459, except: 427.5, 435, 446, 459.0).

**Results:**

The mean age of patients at the time of transplantation was 47.0 ± 14.2 years; 62% were male. 16.5% had suffered some cardiovascular disease prior to transplantation and 9.7% had suffered a cardiovascular event. The mean follow-up period for the patients with cardiovascular event was 3.5 ± 4.3 years. Applying competing risk methodology, it was observed that the accumulated incidence of the event was 5.0% one year after transplantation, 8.1% after five years, and 11.9% after ten years. After applying multivariate models, the variables with an independent effect for predicting cardiovascular events are: male sex, age of recipient, previous cardiovascular disorders, pre-transplant smoking and post-transplant diabetes.

**Conclusions:**

This study makes it possible to determine in kidney transplant patients, taking into account competitive events, the incidence of post-transplant cardiovascular events and the risk factors of these events. Modifiable risk factors are identified, owing to which, changes in said factors would have a bearing of the incidence of events.

## Background

Cardiovascular disease is one of the most common complications after renal transplantation [1]. Although some authors have documented a significant reduction in cardiovascular death after kidney transplantation [2], today cardiovascular disease is still the major known cause of death in kidney transplant patients [3].

Long-term graft failure is secondary to chronic allograft nephropathy, recurrent disease, and death with a functioning graft. Recurrent disease is becoming an important cause of late graft failure and cardiovascular illnesses and neoplasms are the two main causes of death with normal function of the graft in the long-term follow-up of kidney transplant patients [4].

Conventional cardiovascular risk factors such as hyperlipidaemia, hypertension and diabetes are common in transplant recipients, partly because of the effects of immunosuppressive drugs, and are associated with adverse outcomes [5, 6]. Determining the incidence of cardiovascular events after a kidney transplant and the associated risk factors is important to inform physicians of the need of cardiovascular disease screening and prevention as part of the transplant evaluation [7, 8]. On the other hand, an accurate estimation of a patient’s risk of cardiovascular disease could allow identifying people at risk of a cardiovascular event and intervene before they develop the disease.

The study was conducted with the aim determining the incidence of cardiovascular events and the variables associated with the same, employing competing risk methodology to estimate the events of interest.

## Methods

### Study type

Ambispective observational follow-up study within a cohort of renal-transplant recipients. The protocol of this study has been already published [9].

### Research setting

The study included all the kidney transplants performed at the Nephrology Department of Complejo Hospitalario Universitario de A Coruña (Spain) during 1981–2011. This hospital is of reference at a regional level for kidney transplantation and at a national level for combined kidney-pancreas transplantation. It is a 1,382-bed public tertiary care hospital attending a population of nearly 560,000 habitants.

### Study population

An observational prospective follow-up study with a retrospective component during the period 1981–2011 was completed. During said period 2,313 kidney transplants were performed, corresponding to 2,029 patients.

Patients who had received transplants were identified through the hospital’s transplant registry.

### Measurements

For each patient, information included donor and recipient characteristics, patient and graft survival after transplantation. Information about cardiovascular risk factors at the time of transplantation was also collected and post-transplant cardiovascular events were registered. The follow-up period for each patient starts on the day of transplantation and continues until death or last reported contact.

Post-transplant cardiovascular events are defined as the presence of myocardial infarction, invasive coronary artery therapy (coronary balloon angioplasty, stents and bypass surgery), cerebral vascular events (stroke and transient ischemic attacks), new-onset angina, congestive heart failure, rhythm disturbances (ventricular tachycardia, atrial fibrillation and the need for a pacemaker), peripheral vascular disease and cardiovascular disease, death. The cause of death was identified through the medical history and death certificate using ICD9 (390–459, except: 427.5, 435, 446, 459.0).

#### Sample size justification

The sample size (*n* = 2,029) makes it possible to detect as significant HR ≥ 1.305 with a prevalence of exposure to a risk factor of 50% and a censored data percentage of 78% (Security: 95%; Statistical power: 80%).

### Statistical analysis

A descriptive analysis of the variables recorded was performed. Competing risk survival analysis methods were applied to estimate the cumulative incidence of developing events over time from kidney transplantation. These methods allow for the fact that a patient may experience an event which is different from that of interest. These events are known as competing risk events, and may preclude the onset of the event of interest, or may modify the probability of the onset of that event. In particular, a transplanted patient may die or lose the graft without suffering a cardiovascular event. In a Kaplan-Meier estimation approach, these individuals would be treated as censored and would be eliminated from the risk set, leading to misleading results. Using competing risks, the probability of any event happening is partitioned into the probabilities for each type of event.

To determine cardiovascular event-free survival, the primary outcome was the estimation of the probability of cardiovascular disease.

The accumulated occurrence of cardiovascular disease during the follow-up period was estimated using the method proposed by Kalbfleisch and Prentice [10]. The accumulated occurrence of cardiovascular events according to different characteristics was compared using the test proposed by Gray [11]. Finally, in order to identify which characteristics were associated with the risk of cardiovascular events, a multivariate analysis was carried out using the model proposed by Fine and Gray [12]. All of the tests were carried out bilaterally, considering values of *p* < 0.05 as significant. The analyses were carried out using the programmes R 3.2.3 and SPSS 19.0.

## Results

The baseline characteristics of the kidney transplant recipients are given in Table 1.

**Table 1:** Baseline characteristics and cardiovascular risk factors of the kidney transplant recipients

Baseline characteristics	Mean	SD	Median
Age of recipient (years)	46.97	14.2	49.00
Age of donor (years)	42.07	18.7	44.00
Time in renal replacement therapy (months)	31.1	35.0	20.00
	n	%	95% CI
Gender of recipient			
Men	1266/2029	62.4	60.3–64.5
Women	763/2029	37.6	35.5–39.7
Gender of donor			
Men	1329/1982	67.1	65.0–69.1
Women	653/1982	32.9	30.9–35.0
Live donor	83/1939	4.3	3.4–5.2
Pre-transplant substitutive renal therapy			
Haemodialysis	1462/1951	74.9	73.0–76.9
Peritoneal dialysis	489/1951	25.1	23.1–27.0
Prior transplant			
First transplant	1745/2029	86.0	84.5–87.5
Re-transplant	284/2029	14.0	12.5–15.5
DR compatibilities			
0	184/1942	9.5	8.1–10.8
1	1340/1942	69.0	66.9–71.1
2	418/1942	21.5	19.7–23.4
ABO Compatibilities			
0	377/1955	19.3	17.5–21.1
1	791/1955	40.5	38.3–42.7
2	660/1955	33.8	31.6–35.9
3	101/1955	5.2	4.2–6.2
4	26/1955	1.3	0.8–1.9
Type of transplant			
Kidney	1927/2008	95.5	94.6–96.4
Kidney + liver	19/2008	0.94	0.5–1.4
Kidney + pancreas	55/2008	2.73	2.0–3.5
Kidney + heart	12/2008	0.59	0.2–0.9
Kidney + heart + liver	1/2008	0.05	0.001–0.3
Kidney + lung	4/2008	0.20	0.05–0.5
Cardiovascular Risk Factors	n	%	95% CI
Previous cardiovascular disease	328/1983	16.5	14.9–18.2
Obesity (BMI ≥ 30kg/m^2^)	191/1598	12.0	10.3–13.6
Pre-transplant hypertension	1698/1982	85.7	84.1–87.2
Pre-transplant left ventricular hypertrophy	850/1856	45.8	43.5–48.1
Pre-transplant diabetes mellitus	176/1999	8.8	7.5–10.1
Pre-transplant smoker	477/1007	47.4	44.2–50.5

During the study period, a total of 2,313 transplants were performed at the University Hospital Complex in A Coruña, corresponding to *n* = 2,029 patients. The characteristics of the transplants performed and the prevalence of cardiovascular risk factors at the time of transplantation are shown in Table 1.

The graft continued to function at the end of follow-up in 53.3% of transplants, the graft was lost in 30.2% of cases and 16.5% of patients did not survive. Among the causes of death with a functioning graft, the most frequent were infections (28.4%), cardiovascular disease (24.2%) and post-transplant cancer (10.5%). A total of 9.7% of transplantation patients suffered a cardiovascular event during follow-up.

If we analyse the incidence of cardiovascular events and factors associated with the same, it can be observed that the rate of incidence per 1,000 individuals-year of follow-up for cardiovascular events is 22.9 (95% CI: 19.8–26.3), with the mean time elapsed from transplantation to the onset of the cardiovascular event being 3.5 ± 4.3 years. It can be seen that the rate is higher in men than in women (26.8 vs. 16.6 per 1,000 individuals/year; *p* = 0.003) and increases in proportion to the recipient’s age. In patients aged 45 or under, the incidence rate is 12.0 × 1000 individuals/year. Between 46 and 55 years of age, the incidence rate is 26.7 × 1000 individuals/year. In the 56–65 year old age group, it is 3.0 × 1000 individuals/year, and for patients over 65 years of age, it is 69.1 × 1000 individuals/year.

**Fig. 1:**
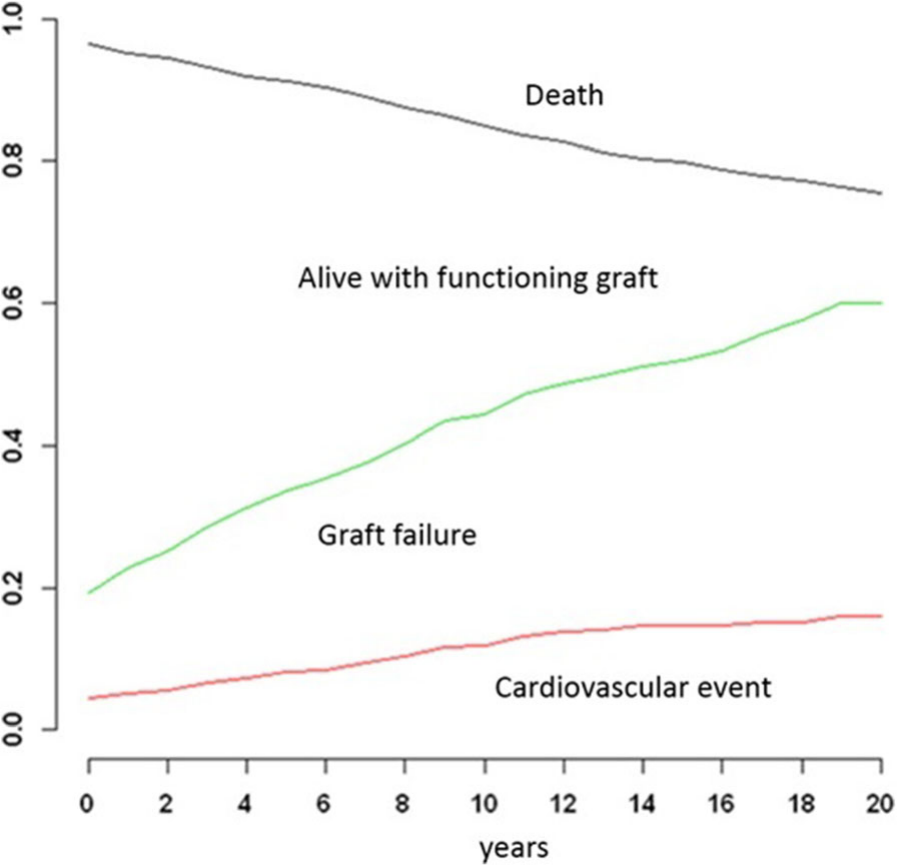
Accumulated incidents according to event (cardiovascular event, loss of graft or death of the patient)

Applying competing risk methodology, it can be observed that the accumulated incidence for cardiovascular events one year after transplantation, after two years, after five years and after ten years, is 5.0, 5.6, 8.1 and 11.9%, respectively (Fig. 1).

The bivariate analysis (Table 2) shows that patients experiencing a cardiovascular event are generally older. At the end of follow-up, 11.2% of the males had suffered a cardiovascular event as opposed to 7.2% of women. The highest percentage of events was in pre-transplantation smokers and post transplantation smokers, in patients with previous cardiovascular disease (cerebral vascular events (stroke and transient ischemic attacks), ischemic heart disease, congestive heart failure, peripheral vascular disease and arrhythmias), in those diagnosed with high post-transplantation blood pressure, in those with left ventricular hypertrophy and post-transplant diabetes. Patients with cardiovascular events had higher levels of glycemic control than patients without cardiovascular events.

**Table 2:** Comparison of recipient kidney transplant according to presence or absence of cardiovascular event

	No Cardiovascular Event	Cardiovascular Event	Univariate Survival Models			
	Mean ± sd	Mean ± sd	Sig,	HR	95% CI (HR)	
Receptor age	46.5 ± 14.4	51.5 ± 11.5	<0.001	1.038	1.026	1.049
Weight (Kg)	67.9 ± 22.5	69.8 ± 13.5	0.015	1.004	1.001	1.007
BMI (kg/m^2^)	25.3 ± 8.3	25.9 ± 3.9	0.050	1.009	1.000	1.019
Proteinuria (g/24h)	0.5 ± 0.9	0.6 ± 1.0	0.086	1.112	0.985	1.256
Hemoglobin (g/dL)	10.0 ± 2.9	9.0 ± 2.7	0.685	0.958	0.777	1.180
Hematocrit (%)	32.7 ± 8.9	31.8 ± 7.1	0.417	1.006	0.992	1.020
Glomerular filtration rate estimated (Cockroft-Gault)	44.5 ± 24.4	43.8 ± 22.9	0.182	0.995	0.988	1.002
Glomerular filtration rate estimated (MDRD)	40.5 ± 25.5	39.9 ± 24.5	0.120	0.995	0.989	1.001
Glomerular filtration rate estimated (CKD-EPI)	50.1 ± 31.5	44.8 ± 28.2	0.062	0.995	0.990	1.001
Systolic blood pressure (mmHg)	141.6 ± 21.3	145.3 ± 22.3	0.230	1.004	0.998	1.010
Diastolic blood pressure (mmHg)	81.9 ± 12.4	81.3 ± 11.6	0.195	0.992	0.981	1.004
Cholesterol (mg/dL)	147.8 ± 40.4	152.3 ± 42.6	0.717	1.001	0.997	1.004
Cholesterol HDL (mg/dL)	40.6 ± 17.8	36.9 ± 15.4	0.580	0.993	0.969	1.017
Cholesterol LDL (mg/dL)	121.2 ± 35.6	111.6 ± 41.9	0.349	0.992	0.976	1.009
Triglycerides (mg/dL)	140.7 ± 73.5	150.8 ± 77.0	0.266	1.001	0.999	1.003
Glucose (mg/dL)	120.1 ± 59.7	124.7 ± 49.7	0.190	1.001	0.999	1.002
	Event in exposed subjects	Event in unexposed subjects	Univariate Survival Models			
	n (%)	n (%)	Sig.	Exp(β) = HR	95% CI (HR)	
Gender (ref:woman)	142 (11.2)	55 (7.2)	0.002	1.615	1.182	2.205
Previous cardiovascular illness	65 (19.8)	131 (7.9)	<0.001	2.972	2.206	4.004
Pre-transplantation smokers	65 (13.1)	33 (6.5)	<0.001	2.819	1.842	4.316
Post-transplantation smokers	22 (15.0)	69 (8.2)	<0.001	2.323	1.434	3.763
Pre-transplantation hypertension	165 (9.7)	30 (10.6)	0.472	1.154	0.781	1.707
Post-transplantation hypertension	193 (10.3)	4 (3.6)	0.037	2.872	1.067	7.731
Diabetes	67 (14.0)	129 (8.5)	<0.001	1.820	1.360	2.430
Left ventricular hypertrophy	109 (13.9)	75 (7.0)	<0.001	2.150	1.602	2.886

After modelling the incidence of cardiovascular events employing specific competing risk techniques, with the patient’s death or graft loss being those events competing with the presence of the event of interest, adjusting for different factors, it can be seen that those variables with a bearing on the presence of cardiovascular events subsequent to kidney transplantation are male gender, age of the recipient, prior cardiovascular disease, pre-transplant smoking and diabetes (Table 3).

**Table 3:** Multivariate competing risk regression model of a cardiovascular event after kidney trasplantation

	β	se (β)	*p*	HR	95% CI	
Gender (ref: woman)	0.589	0.288	0.041	1.80	1.02	3.17
Receptor age	0.021	0.007	0.005	1.02	1.01	1.04
Previous cardiovascular illness	0.655	0.221	0.003	1.92	1.25	2.97
Pre-transplant smoking	0.558	0.242	0.021	1.75	1.09	2.81
Post-transplant diabetes mellitus	0.585	0.212	0.006	1.79	1.18	2.72

## Discussion

In this study, data were collected on 2,029 kidney transplant patients, with a mean age of 47.0 ± 14.2 years, and 62.4% of which were men. A cardiovascular event was suffered by 9.7% of these patients. The accumulated incidence of a cardiovascular event in the presence of competing risks was 5.0% in the first year, 6.6% after three years, 8.1% after five years and 11.9% after ten years. These results are consistent with those published in multicentre studies, including data from transplanted patients from North America, Europe and countries on the Pacific coast, in which the accumulated incidence of coronary events and the accumulated incidence of myocardial infarct after kidney transplant is estimated [13, 14].

Various epidemiological studies have identified the factors associated with an increase in the probability of falling ill or dying owing to cardiovascular disease after kidney transplantation [15–17]. They also test whether the risk factors identified for the general population also increased the risks in kidney transplant patients and to what extent. These studies would suggest that the cardiovascular risk factors for the general population (e.g. high blood pressure, hyperlipidaemia and smoking) are predictive of events in the transplanted population. Diabetes doubles the risk of events in men and triples the risk in women with respect to that estimated for the general population; moreover, it is shown that the episodes of acute rejection during the first year after transplantation are associated with a greater risk [15, 16]. In the study by Weiner, D. E. [17] the factors associated significantly with an increased risk of cardiovascular disease in transplanted patients were old-age, prior cardiovascular disease, diabetes, smoking, systolic and diastolic blood pressure and low BMI and glomerular filtration rates estimated with the CKD-EPI formula lower than 45ml/min/1.73m^2^. Other traditional risk factors in the general population, such as gender, LDL Cholesterol and triglycerides, were not significant.

For cerebrovascular disease (ischaemic cerebrovascular accident, haemorrhagic cerebrovascular accidents and transient ischaemic attacks) after kidney transplantation, the risk factors were age, pre-and post-transplant smoking, diabetes, high blood pressure, obesity and coronary comorbidity [5].

The results of our study are consistent with those in the literature. After performing survival models, we have identified the following as predictors of cardiovascular events: male gender, age of recipient, the presence of prior cardiovascular disease, pre-transplant smoking and post-transplant diabetes. The presence of these factors along with the higher age of the recipient increases the risk of cardiovascular events.

Here we should stress the importance of complications related with immunosuppressant treatment, of which diabetes mellitus is perhaps most important, owing to the vascular problems it gives rise to [18–21]. We believe that the prevention of new-onset diabetes is a key strategy for reducing post-transplant cardiovascular mortality. The individualisation of immunosuppression guidelines (selection of calcineurin inhibitor and the steroid dosage) may be an effective measure for controlling the incidence of post-transplant diabetes and modifying the cardiovascular risk in this patient group.

Among the limitations of the study, we could point out that with a view to minimising the selection bias, information was collected on all transplants performed during the study period. There were no significant differences in information losses among patients presenting the event of interest or not. In order to minimise any information biases, mean values of the baseline measurements closest in time were calculated. Left ventricular hypertrophy was diagnosed by ECG. As opposed to previous studies—based on the retrospective analysis of records, which only contain information prior to transplantation and from short-term follow-up—this study provides long-term information on clinical and analytic parameters and with regard to the treatment of kidney transplant recipients. Although the information was gathered from hospital medical records, which could result in an information bias, the characteristics of these patients mean that they are subject to much more exhaustive follow-up than is habitual, not only during the period immediately after transplantation, but throughout the entire follow-up period. Thus, during the first year after kidney transplantation, patients were seen every 3 months, every 6 months in the 10 years following a transplant, and once a year after the first 10 years of follow-up. The study included a large cohort of patients with a long period of post-transplantation follow-up, which has enabled us to obtain valid results on the long-term results of kidney transplantation. Among the confounding biases, it should be noted that there are risks factors which were not included, information in relation with the heart failure according to the functional state was not available and also the level of HbA1c expressing glicemic control in diabetics was not included. Treatments, which modify the variables studied, were not included. In order to control the confusion, we have employed multivariate regression techniques. The population at risk changes over time in a population of survivors due to the occurrence of competing events. It is possible that the presence of competing events has an impact on the effect estimates over time.

The majority of the studies published in the literature employ the habitual survival analysis techniques, assuming that there is only one event of interest, and that censoring is not informative; i.e., that if we monitored censored patients, they would have the same rate for the event as non-censored patients [22]. In practice these assumptions are not always correct; generally speaking, an individual may experience more than one type of event, or experience a type of event which hinders or modifies the probability of observing the event of interest. Competing risk survival analysis enables us to determine the factors associated with the incidence of a specific event. Fine & Gray [12] modified Cox’s proportional risk model to take into account competing risks.

It is becoming progressively easier to secure comprehensive data sets with full follow-up; consequently, the need to apply mathematical methodologies focused on the precise type of analysis is also increasing. Owing to the foregoing, our principal aim must be to apply specific techniques, such as competing risks, from the design phase of the study up to the interpretation of the results obtained.

## Conclusions

This study has made it possible to determine, in a cohort of kidney transplant recipients, and taking into account competing events, the post-transplant incidence of said events and the risk factors associated with the same. Modifiable risk factors have been identified, owing to which, said factors would have a bearing of the incidence of cardiovascular events in this patient group.

## Abbreviations

BMI: Body mass index
CKD-EPI: Chronic kidney disease epidemiology collaboration
HR: Hazard ratio
